# Apathy in Cerebral Small Vessel Disease Stroke Is a Predictor of Quality of Life, Mood and Distress in Both Patients and Carers

**DOI:** 10.1002/gps.70178

**Published:** 2025-12-03

**Authors:** Oriane E. Marguet, Claudia Pallucca, Robin Morris, Hugh S. Markus

**Affiliations:** ^1^ Department of Clinical Neurosciences University of Cambridge Cambridge UK; ^2^ Kings' College Institute of Psychiatry Psychology and Neuroscience London UK

**Keywords:** apathy, caregivers, cerebral small vessel disease, stroke

## Abstract

**Objective:**

Apathy occurs in one‐third of patients after stroke and is particularly common in small vessel disease (SVD) stroke. Apathy is known to be associated with reduced ability to engage in everyday life activities, including neurorehabilitation. Apathy has been associated with increased caregiver burden and distress in other neurological diseases, but there is little data on stroke. We aimed to evaluate whether distress is higher and quality of life (QoL) lower in apathetic stroke patients and their caregivers.

**Methods:**

We conducted a cohort study on patients with symptomatic SVD and their caregivers. We recruited 40 patient‐caregiver pairs (20 with apathy and 20 without) and assessed apathy, patient disability, QoL, cognition and depression. We assessed apathy‐related caregivers' distress, caregiver burden, mood and quality of life. Statistical tests were performed to compare the apathetic and control groups. Univariate correlations and multiple linear regressions were used to assess relationships between apathy and measures of quality of life, burden, distress and mood.

**Results:**

Apathy was a strong predictor of caregiver distress (*β* = 0.11, *p* < 0.001), burden (*β* = 0.7, *p* < 0.001) and depression (*β* = 0.12, *p* < 0.001). In contrast, physical disability was not an independent predictor of caregiver burden. Apathy was also a strong predictor of patient QoL (*β* = −0.67, *p* < 0.001) and depression (*β* = 0.49, *p* < 0.001).

**Conclusion:**

Apathy is a major independent predictor of caregiver burden. Caregivers of apathic patients experience increased distress, burden and lower mood. Patients with apathy also experience lower mood and worse QoL. The major effect of apathy on caregivers suggests targeting them should be an important component of the treatment of apathy in patients with stroke.

## Key Points

- Apathy in cerebral small vessel disease (SVD) affects patients' quality of life
- Apathy in SVD has a significant impact on caregivers' mood, distress and burden
- Apathy is the main predictor of distress in caregivers of patients with SVD
- Therapeutic strategies should be developed to target both patients and caregivers

**Abbreviations**
AES (‐C, ‐I, ‐S): Apathy Evaluation Scale (Clinician, Informant, Self‐report)
BDI: Beck Depression Inventory
BMET: Brief Memory and Executive Test
HADS: Hospital Anxiety and Depression Scale
NPI: Neuropsychiatric Inventory
QoL: Quality of Life
SF‐36 (M/PCS): Thirty six‐Item Short Form Survey (Mental/Physical Component)
SVD: Cerebral Small Vessel Disease
ZBI: Zarit Burden Interview

## 1 Introduction

Apathy is a behavioural syndrome that occurs in one‐third of patients after stroke [1, 2]. It is characterised by loss of motivation, combined with reduction in goal‐directed behaviours (GDB) relative to an individual's previous level of functioning. Post‐stroke patients with apathy experience greater functional impairment and demonstrate slower recovery times [2, 3]. However, despite its high prevalence and an impact on outcomes after stroke there are few effective treatments [4]. No drug therapies have been shown to be effective, and while antidepressants are sometimes given, apathy is distant and dissociable from depression, and anti‐depressants have not been shown to reduce apathy after stroke [5, 6].

Alternative approaches include neurorehabilitation or behavioural management and therapy. In relation to stroke, a number of more intensive behavioural approaches have been proposed including goal setting with an emphasis on planning future goals and evaluating success to help re‐establish GDB, problem‐solving, wherein a patient selects an activity and makes a plan to achieve it while self‐monitoring the process and outcome, and behavioural activation [2]. However, an important consideration is whether the apathy or loss of motivation prevents the patient from engaging sufficiently in their intervention. Engagement is also likely to be influenced by the patient's insight into their apathy, and the perceived benefit in terms of their quality of life (QoL), with some patients appearing unconcerned by their apathy syndrome [1, 7].

The effect of apathy as a syndrome is not confined to the person. In other neurological diseases, apathy has been shown to have a major effect on their caregivers, including partners and family. Caregivers of people with stroke may experience high emotional burden, this in turn affecting their mood and quality of life [8, 9]. Moreover, there is evidence that the caregiver's emotional state correlates with the patient's recovery, and the psychological well‐being of stroke survivor caregivers is correlated with the functional and physical recovery of the patient [10, 11]. Studies have shown that apathy in particular is associated with increased burden and distress in various neurological diseases including for example, dementia [12, 13] and motor neuron disease [14]. Nevertheless, although there exist some studies of associations between apathy and patient distress or caregiver burden in stroke, they remain scarce [15, 16, 17] Exploring this association may have implications for neurorehabilitation of people with stroke, including working with the caregiver to improve their experience of caregiving.

Whilst apathy is found across the different types of stroke, it is particularly common in the small vessel disease stroke subtype (SVD). SVD causes a quarter of all ischemic strokes and represents the most common cause of vascular cognitive impairment [18]. It can be sporadic or familial, the most common form of the latter being CADASIL. Apathy has been reported as a common symptom in both sporadic and familial SVD [2, 19]. In SVD, apathy can present in the absence of major physical difficulties but nevertheless can be a major cause of disability [2]. It has been suggested that apathy occurs due to white matter tract disruption causing a ‘network disorder’ [20]. Whilst large infarcts can cause a variety of neurological or neuropsychological features, the damage caused by SVD tends to be more homogenous and so potentially more straightforward in terms of modelling of associations between the pathophysiology, the behavioural consequences and association with psychosocial sequelae such as the effect on the caregiver. Accordingly, we recruited patients with symptomatic SVD, both sporadic and familial, and measured their levels of apathy in conjunction with focussing on the experience of their caregivers, with markers of distress, burden, emotional wellbeing and quality of life.

## 2 Materials and Methods

### 2.1 Study Design

We conducted a cross‐sectional study in a comprehensive stroke service and recruited 40 patient‐caregiver pairs. Patients and their caregivers were invited to a single visit which included cognitive testing, mood and neuropsychiatric assessment. Recruitment took place between May 2022 and November 2024.

### 2.2 Ethical Approval

The study was approved by the West of Scotland Research Ethics Committee (REC reference: 22/WS/0010) and written informed consent was obtained from all patients and caregivers.

### 2.3 Recruitment

Patients with both sporadic and familial SVD (CADASIL) were eligible. For sporadic SVD, patients with symptomatic cerebral small vessel disease defined as a clinical lacunar stroke syndrome with an anatomically corresponding MRI lacunar infarct were recruited from the Addenbrooke's Hospital Stroke Unit and stroke outpatient clinics. We only included patients with a corresponding MRI‐confirmed lacunar infarct to ensure diagnostic specificity for symptomatic SVD and avoid heterogeneity from other stroke subtypes. Patients with CADASIL were recruited from a specialist clinic in the same stroke service. To aim for equal number of apathetic and non‐apathetic patients, clinicians across the service were asked to identify patients with SVD and a history suggestive of apathy. Patients were also identified with SVD without obvious apathetic symptoms. Additional inclusion criteria were: fluent in English to allow cognitive and other assessments. Exclusion criteria were: a clinical diagnosis of dementia, aphasia, and unable to participate, and any another major central nervous system or psychiatric disorder diagnosed by a clinician. Inclusion also required that there was involvement of a caregiver, these defined by being a spouse, family member or relative able to act as an informant; able to give informed consent; ≥ 18 years old; and fluent in English.

### 2.4 Sample Size Calculation

This was based on a previous study investigating the effect of apathy in Parkinson's disease which found significantly greater burden in caregivers of participants with apathy. Their sample size included 22 apathetic and 28 non‐apathetic patients, each group with their respective caregivers [21]. An a priori power analysis was conducted based on the data from that study. Their effect size was 0.9, considered to be large using Cohen's criteria. With a significance criterion of *α* = 0.05 and power = 0.80, the minimum sample size needed was 38 for a two‐sample *t*‐test. Therefore, we aimed to recruit 40 pairs of patients‐carers to be split in two groups (20 with apathy, 20 without).

### 2.5 Data Collection

Patients: Apathy was then assessed using the Apathy Evaluation Scale—Clinician report (AES‐C) [22], with apathy defined as an AES‐C score ≥ 34. The AES‐C is considered the most accurate version of the AES, with an interrater reliability of 94%. During the visit, demographic information (including self‐reported ethnicity), medical history and stroke details were collected. Current depressive symptoms were assessed using the DSM‐5 criteria, and patients with either major depressive disorder, minor depressive disorder, or with prescriptions of psychoactive medication for depression were recorded as ‘currently depressed’. Past history of depression was also recorded. Patient disability was evaluated with the Modified Rankin Scale (mRS), which assesses disability ranking from 0 (no symptoms) to 5 (severe disability) and 6 (deceased) [23]. Cognition was tested using the Montreal Cognitive Assessment (MoCA) [24] and the Brief Memory and Executive Test (BMET). The BMET is a brief cognitive test that has been designed to be sensitive to the ‘subcortical’ pattern of cognitive impairment seen in SVD [25]. Depression was measured using the Beck Depression Inventory (BDI), a self‐scored inventory assessing symptoms of depression such as guilt, low mood, fatigue and anhedonia. A high score indicates worse mood [26]. QoL was assessed with the 36‐Item Short Form Survey (SF‐36), a QoL questionnaire that consists of a variety of questions on physical and mental health [27]. The questions are then weighed differently into forming two components, the MCS and PCS, that were considered separately. A higher score indicates better QoL.

Caregivers: Demographics were collected (including self‐reported ethnicity); apathy‐related and depression‐related distress were measured using the Neuropsychiatric Inventory (NPI) [28], caregiver burden with the Zarit Burden Interview (ZBI) [29], anxiety and depression with the Hospital Anxiety and Depression Scale (HADS) [30], and QoL with the SF‐36. The NPI is an informant‐based interview that assesses a range of psychiatric symptoms over three aspects: their frequency, their severity, and the distress they bring to the informant via the NPI Caregiver Distress Scale, one subscale of the test [31]. A higher score indicates higher caregiver distress. The ZBI is a caregiver‐based interview commonly used to assess caregiver burden, with higher scores associated with higher burden. Finally, the HADS is a questionnaire separated into two scales, anxiety and depression, commonly used in clinical research. Both scales are considered individually, and higher scores are associated with worse mental health.

### 2.6 Statistical Analysis

All statistical analyses were performed using R 4.2.3 [32] and results were considered significant when *p* ≤ 0.05. Our significance thresholds were indicated as *(*p* ≤ 0.05), **(*p* ≤ 0.01) and ***(*p* ≤ 0.001). Demographics, cognition scores, measures of QoL, distress, caregiver burden and mood were compared between the apathetic and control groups. Continuous variables were assessed with independent *t*‐tests if normally distributed and Mann‐Whitney U tests alternatively. Categorical variables were compared with χ^2^‐tests or Fisher's exact tests in case of small group size.

In the total cohort, correlations between the AES‐C score, and caregiver PCS and MCS, ZBI, NPI (apathy‐related distress), NPI (depression‐related distress), HADS (anxiety scale), HADS (depression scale), patient PCS and MCS, and patient BDI were assessed using Spearman's correlation. Multiple linear regression models were then built to further assess the relationships between these variables after correcting for age and sex of patients and caregivers, mRS, and total BMET score. Furthermore, significant multivariate associations were analysed in causal mediation analysis using the R mediation package 4.5.0 [33]. To further assess predictors of caregiver burden, a multiple linear regression model was built to predict score on the ZBI and reduced to the minimal model using backward elimination.

### 2.7 Missing Data

Two participants did not complete the MoCA and one did not complete the BMET due to fatigue during the visit, resulting in 38 pairs with MoCA test results and 39 with BMET results. One caregiver did not complete the HADS, the ZBI and the SF‐36 due to misunderstanding the instructions, resulting in 39 pairs with available data on these questionnaires. Due to their low proportion, missing data were treated as unavailable (NA) and were excluded from the analyses on a case‐by‐case basis for specific variables where data were missing. Analyses were conducted on the remaining participants with complete data for the respective variables.

## 3 Results

### 3.1 Cohort Characteristics

**FIGURE 1:**
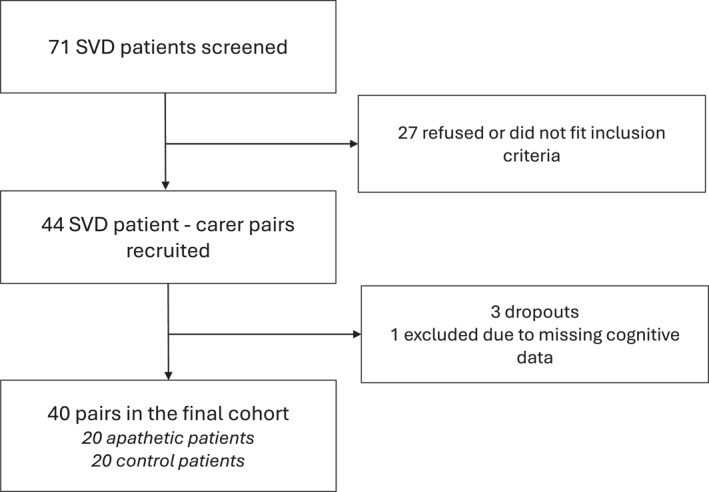
Recruitment flow chart. Out of 71 patients screened, 44 were eligible for the study together with their caregivers. Four patient‐carer pairs were excluded after their visit due to loss of consent or significant missing data. The final cohort consisted of 40 pairs including 20 patients with apathy and 20 without.

Seventy one patients were screened. 27 refused to participate or did not fit the inclusion criteria. 44 pairs of patients and their caregivers were recruited of which 3 decided not to proceed and one was excluded due to refusal to complete the cognitive tests. Our final cohort consisted of 40 patient‐caregiver pairs. These 40 pairs were split into whether the patients had apathy as defined by the AES‐C score being ≥ 34, this group termed apathetic patients (*n* = 20), and the remainder not achieving this criterion termed controls (*n* = 20). A recruitment flow chart can be found in Figure 1.

The apathy and control groups did not differ in age, sex, education, or ethnicity, for patients or for caregivers (Tables 1 and 2). Patients from the apathetic group scored significantly worse on the BMET executive scale, BDI, mRS and SF‐36 MCS. On the other hand, the groups showed no significant difference on the MoCA and SF‐36 PCS score. The discrepancy between the BMET executive scale and the MoCA might be due to the design of the BMET, specifically developed to capture subcortical executive dysfunction characteristic of SVD, and therefore possibly more sensitive than the MoCA to cognitive differences amongst our participants [25]. Caregivers of apathetic patients had significantly higher scores for anxiety, depression, apathy‐related distress, depression‐related distress, and caregiver burden. We found no difference in caregivers QoL scores.

**TABLE 1:** Characteristics of the study sample: patients; *p*‐values are for the analysis of apathy versus control groups. A 95% confidence interval (CI) is provided for numerical data.

Variable	Cohort (*n* = 40)	Apathy (*n* = 20)	Control (*n* = 20)	*p*‐value	95% CI
Age	66.35 ± 11.79	65.9 ± 11.50	66.8 ± 12.36	0.81	[−0.69, 0.55]
Sex (*n*, % male)	28 (70)	16 (80)	12 (60)	0.3	—
Ethnicity (*n*, %)				1	—
Asian	3 (7.5)	2 (10)	1 (5)		
White	37 (92.5)	18 (90)	19 (95)		
Years education	14.49 ± 3.75	14.18 ± 4.04	14.8 ± 3.50	0.38	[−0.48, 0.19]
CADASIL (*n*, %)	19 (47.5)	12 (60)	7 (36.8)	0.21	—
Diagnosis of depression (past & current) (*n*, %)	14 (35)	11 (55)	3 (15)	**0.02** ^a^	—
Diagnosis of depression (current) (*n*, %)	12 (30)	10 (50)	2 (10)	**0.016** **^a^**	—
mRS median	1	1.5	0	**0.018** **^a^**	[0.09, 0.67]
MoCA total^a^	23.39 ± 4.89	21.72 ± 5.94	25.25 ± 3.007	0.053	[−1.33, 0.009]
BMET total^b^	12 ± 3.56	11.11 ± 4.19	13.5 ± 2.4	0.077	[−0.61, 0.03]
BMET executive	6.74 ± 1.82	6.05 ± 1.82	7.4 ± 1.14	**0.013** ***^a^***	
BMET memory	5.56 ± 2.23	5.11 ± 2.54	6 ± 1.86	0.232	
AES clinician	37.28 ± 12.3	47.7 ± 8.05	26.9 ± 4.35	< 0.001***	[2.17, 4.22]
AES self‐rated	37.6 ± 11.34	45.6 ± 8.81	26.9 ± 4.35	< 0.001***	[0.65, 0.90]
AES informant	38.1 ± 12.26	46.65 ± 10.14	27.65 ± 9.49	< 0.001***	[1.21, 2.75]
BDI	9.95 ± 7.78	13.4 ± 8.33	6.5 ± 5.45	**0.004** **^b^**	[0.31, 1.64]
SF‐36 (mental)	47.72 ± 10.73	42.86 ± 11.83	52.57 ± 6.84	**0.003** **^b^**	[−0.77, 0.48]
SF‐36 (physical)	36.03 ± 11.32	34.42 ± 10.08	37.64 ± 12.5	0.38	[−0.58, 0.67]

*Note:* Significant *p*‐values are indicated in bold. Numerical data is presented as mean ± standard deviation; categorical data as number of observations and percentage.

^a^ The MoCA total score was available for 38 participants out of 40 as explained in the Methods—Missing data.

^b^ The BMET total score was available for 39 participants out of 40 as explained in the Methods—Missing data.

**TABLE 2:** Characteristics of the study sample: caregivers; *p*‐values are for the analysis of the apathy versus control groups. A 95% confidence interval (CI) is provided for numerical data.

Variable	Cohort (*n* = 40)	Apathy (*n* = 20)	Control (*n* = 20)	*p*‐value	95% CI
Age	63.43 ± 12.12	62.6 ± 12.98	65.9 ± 10.76	0.67	[−0.75, 0.49]
Sex (*n*, % male)	10 (25)	3 (15)	7 (35)	0.27	—
Ethnicity (*n*, %)				1	—
Asian	4 (10)	2 (10)	2 (10)		
White	36 (90)	18 (90)	18 (90)		
Years education	13.78 ± 3.1	13.3 ± 2.79	14.25 ± 3.31	0.33	[−0.50, 0.18]
Relationship with patient (*n*, %)				0.6	—
Child	2 (5)	1 (5)	1 (5)		
Friend	1 (2.5)	0 (0)	1 (5)		
Partner	38 (92.5)	19 (95)	18 (90)		
NPI apathy‐related distress	1.28 ± 1.74	2.5 ± 1.73	0.05 ± 0.22	< 0.001***	[0.60, 0.89]
NPI depression‐related distress	1.43 ± 1.66	2.3 ± 1.77	0.6 ± 1.05	0.002**	[0.22, 0.74]
ZBI^a^	17.77 ± 13.7	25.5 ± 14.07	9.63 ± 7.14	< 0.001***	[0.44, 0.83]
HADS depression^a^	4.08 ± 3.40	5.5 ± 3.7	2.58 ± 2.29	0.008**	[0.17, 0.72]
HADS anxiety^a^	5.82 ± 4.14	6.95 ± 4.1	4.63 ± 3.95	**0.047** **^a^**	[0.03, 0.64]
SF‐36 (Mental)^a^	45.26 ± 11.29	44.45 ± 11.91	46.11 ± 10.86	0.65	[−0.77, 0.48]
SF‐36 (Physical)^a^	42.38 ± 10.08	42.60 ± 10.48	42.15 ± 9.93	0.89	[−0.58, 0.67]

*Note:* Significant *p*‐values are indicated in bold. Numerical data is presented as mean ± standard deviation; categorical data as number of observations and percentage.

^a^ These variables were available for 39 participants out of 40 as explained in the Methods—Missing data.

To avoid any impact of diagnosis of CADASIL versus sporadic SVD on results, we also compared the characteristics of patients with CADASIL (*n* = 19) and sporadic SVD (*n* = 21). The results are available in Supporting Information S1: Table S1. CADASIL patients were younger as expected. There were no significant differences in other variables such as cognitive scores, depression history, BDI and SF‐36. AES‐I and AES‐S average scores were slightly higher in the CADASIL group; however, there was no significant difference in the AES‐C score which was used for further analysis.

### 3.2 Relationship of Apathy With Burden, Distress, QoL and Mood for Caregivers

In the full cohort (*n* = 40), univariate correlations indicated strong associations between AES‐C score and apathy‐related distress (*r*_*s*_ = 0.83, *p* < 0.001), depression‐related distress (*r*_*s*_ = 0.53, *p* < 0.001), burden (*r*_*s*_ = 0.55, *p* < 0.001), anxiety (*r*_*s*_ = 0.37, *p* = 0.002) and depression (*r*_*s*_ = 0.47, *p* = 0.003) in caregivers. No significant relationship was found between AES‐C and measures of QoL. When controlling for patient age, caregiver age, patient sex, caregiver sex, BMET score and mRS in multiple linear regression models, AES‐C remained a major predictor of apathy‐related distress (*β* = 0.11, *p* < 0.001), depression‐related caregiver distress (*β* = 0.09, *p* < 0.001), caregiver burden (*β* = 0.7, *p* < 0.001), and depression (*β* = 0.12, *p* = 0.02), as shown on Table 3. No covariate had statistical significance. When correcting for multiple comparison with a Bonferroni correction and adjusting the significance threshold as *p* < 0.01, AES‐C remained a significant predictor of all variables cited above apart from depression.

**TABLE 3:** Multiple linear regressions assessing apathy in relationship to caregivers' outcomes.

Outcome variable	Unstandardised coefficient (*β*) of predictor; [95% CI]	*p*‐value
NPI distress apathy	AES‐C 0.11 [0.09; 0.146]	< 0.001***
NPI distress depression	AES‐C 0.09 [0.05; 0.13]	< 0.001***
ZBI	AES‐C 0.7 [0.34; 1.06]	< 0.001***
HADS anxiety	AES‐C 0.11 [−0.014; 0.23]	0.07
HADS depression	AES‐C 0.12 [0.021; 0.22]	0.02*

*Note:* Significant *p*‐values are indicated in bold.

To avoid any impact of patient depression on caregiver burden, we repeated the linear models additionally correcting for current diagnosis of major or minor depressive disorder in the patient. The results, available in Supporting Information S1: Table S2, kept their significance similar to in the previous linear models, although it should be acknowledged that the *p*‐value for ZBI decreased from *p* < 0.001 to *p* = 0.002, showing a small influence of depression on overall carer burden.

To further investigate what variables could predict caregiver burden, we constructed a full linear regression model and reduced it to its minimal model via backward stepwise elimination. The only variable that remained a predictor of caregiver burden was AES‐C score (*β* = 0.73, *p* < 0.001).

### 3.3 Relationship of Apathy With Patient QoL and Mood

Next, we assessed the relationship of AES‐C with patient's SF‐36 MCS, SF‐36 PCS and BDI. There were strong univariate correlations between AES‐C and patient MCS (*r*_*s*_ = −0.52, *p* < 0.001) and BDI (*r*_*s*_ = 0.53, *p* < 0.001). No significant relationship was observed with PCS. The associations identified remained significant in multiple linear regression models correcting for patient age, caregiver age, patient sex, caregiver sex, mRS and BMET as summarised in Table 4. We again repeated the linear models additionally correcting for current diagnosis of major or minor depressive disorder in the patient; this did not impact the significant of our results as shown in Supporting Information S1: Table 3. When correcting for multiple comparison with a Bonferroni correction and adjusting the significance threshold as *p* < 0.025, AES‐C remained a significant predictor of MCS and BDI.

**TABLE 4:** Multiple linear regressions assessing apathy in relationship to patients' outcomes.

Outcome variable	Unstandardised coefficient (*β*) of predictor and significant covariates; [95% CI]	*p*‐value
SF‐36 MCS	AES‐C −0.67 [−0.92; −0.41]	< 0.001***
BDI	AES‐C 0.49 [0.31; 0.67]	< 0.001***
	BMET total 0.97 [0.32; 1.62]	0.013*

*Note:* Significant *p*‐values are indicated in bold.

**FIGURE 2:**
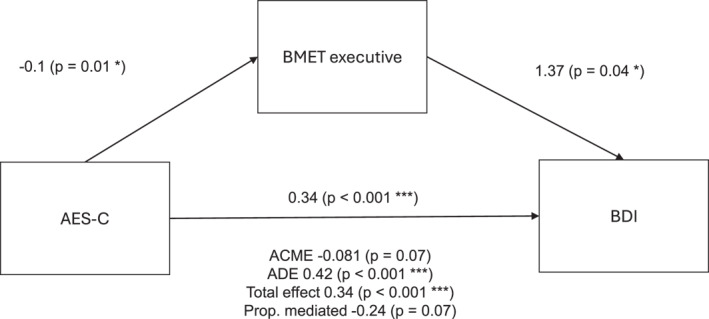
Mediation analysis of AES‐C, BMET and BDI. The relationship between the score from the AES‐C and the score from the BDI is partially, negatively mediated by the executive function score from the BMET (proportion mediated = −0.24).

Since the BMET total score was identified as a significant covariate in the model predicting BDI score, a mediation analysis was performed to further explore the relationship between these variables. It showed that the total BMET score has a partial negative mediating effect on the relationship between AES‐C and BDI, as summarised in Figure 2.

## 4 Discussion

Our results indicate that caregivers of apathetic patients with SVD have higher caregiver burden, caregiver distress, and depression. In univariate correlations, apathy score on the AES‐C had a strong relationship with caregiver distress, burden, anxiety and depression; apathy score was the strongest predictor of caregiver burden in a linear regression model and was a much more important predictor than disability or the patient's mood. Additionally, our data shows that apathetic patients with SVD have a lower mood and lower QoL. Taken together, this confirms that similarly to what is seen in other neuropsychiatric disorders, apathy has a strong impact on both patients with SVD and their caregivers. This reinforces the importance of ensuring that both patient with apathy and their caregivers receive appropriate support.

This study aimed to clarify whether patients or caregivers are more affected by symptoms of apathy: it is clear from the results of our analyses that apathy impacts both these groups severely. As well as highlighting this association, these results may inform the development of treatment approaches for apathy in stroke patients. The increased caregiver distress suggests that targeting treatment on caregivers might produce positive results to alleviate caregiver burden and, in turn, improve patients' wellbeing. Notably, it would be important to look out for depression in caregivers of patients with high apathy. Strategies to reduce caregiver burden have previously been suggested in the domain of stroke and dementia, such as the *Preparing is Caring* or *Care Ecosystem* interventions [34, 35]. These focus on several aspects, and notably prioritise an increased support system for caregivers by providing access to a helpline, guides on how to behave in different scenarios, or supervised sessions with a healthcare specialist. In addition, specific behavioural interventions targeted at caregivers to understand and manage the patients' apathy are being evaluated in other neurological disorders [36].

Of note, there was no relationship between AES‐C and scores on the SF‐36 for caregivers, despite caregivers of apathetic patients showing significantly worse mood and distress. This raises the question of how the SF‐36 questionnaire defines QoL as compared to questionnaires assessing other parameters such as depression or anxiety. We hypothesise that the SF‐36 questionnaire might not be precise enough to differentiate between the caregivers in our study as they have an overall similar profile as caregivers of stroke survivors or CADASIL participants. The presence of apathy or not in the person they care for might not be enough to impact QoL in a way that could be detected by this test, in opposition to more precise tests investigating aspects of mood or specifically designed for caregiver burden. Additionally, both the MCS and the PCS of the SF‐36 are constructed based on the same questions, meaning that physical health also impacts the mental score and inversely. Keeping this in mind, this could explain why this test might be less sensitive to the mental health of caregivers who would be in good physical health. For future studies, a different QoL scale might be selected such as the Euroqol 5D‐5 L. This widely used questionnaire consists of 5 domains separately assessed (mobility, self‐care, usual activities, pain/discomfort, anxiety/depression) as well as a visual analogue scale where the participant can estimate their overall health. Despite the shorter length of this questionnaire compared to the SF‐36, the fact that all domains can be assessed separately might give a better understanding of specific aspects of QoL. For example, in the case of caregiver burden, we might not focus on their mobility but rather on their anxiety/depression scale and visual analogue scale. The Euroqol visual analogue scale was notably used in a study investigating QoL of caregivers of stroke patients [37].

In our study, we chose to select participants with sporadic SVD on the basis of a lacunar infarct confirmed on MRI. There are other imaging features that occur in SVD, such as white matter hyperintensities; and we acknowledge that similar results would likely be obtained in participants with apathy without lacunar stroke but with other MRI markers of SVD. However, some markers such as white matter hyperintensities also occur in other diseases, and therefore we required a lacunar infarct to ensure that the underlying pathology of the participants is likely SVD.

This study has a number of strengths. This study focuses on a cohort of patients with the SVD stroke subtype and their caregivers. There has been little research conducted on QoL and caregiver burden for this specific population. We studied both sporadic and familial SVD and showed a similar pattern across both types of SVD. It also has limitations. This study is limited in range of ethnicity as the majority of patients and caregivers were from a White background, which might not reflect appropriately the distress and burden encountered by other ethnic groups, which should be explored in future studies. Finally, it should be noted that there might be some overlapping components between the AES‐C and the BDI, notably two questions about decision‐making and interest in seeing people; although the questionnaires focus on two different conditions, this intersection might contribute to the relationship between AES‐C and BDI scores that was observed. Despite these limitations, this study provides a methodology for future research on apathy and QoL in cerebrovascular diseases.

## 5 Conclusion

In conclusion, our study demonstrates the major burden that apathy places on caregivers and suggests targeting them should be an important component of the treatment of apathy in patients with stroke.

## Funding

This research was funded by a priority programme grant from the Stroke Association (PPA 2015/02) Infrastructural support was provided by the Cambridge British Heart Foundation Centre of Research Excellence (RE/18/1/34212), and Cambridge University Hospitals NIHR Biomedical Research Centre (BRC‐1215‐20014). The views expressed in this publication are those of the authors and not necessarily those of the NIHR, NHS, or UK Department of Health and Social Care.

## Ethics Statement

All human and animal studies have been approved by the appropriate ethics committee and have therefore been performed in accordance with the ethical standards laid down in the 1964 Declaration of Helsinki and its later amendments.

## Consent

All participants gave their informed consent prior to their inclusion in the study.

## Conflicts of Interest

The authors declare no conflicts of interest.

## Supporting information


Supporting Information S1

## Data Availability

The authors have nothing to report.
