# Supplementary material for: Apathy in Cerebral Small Vessel Disease Stroke Is a Predictor of Quality of Life, Mood and Distress in Both Patients and Carers
Source: Int J Geriatr Psychiatry. 2025 Dec 3;40(12):e70178. doi: 10.1002/gps.70178 (PMC12676198; doi:10.1002/gps.70178)
Supplement: Supplementary file 1 — Supporting Information S1 [file GPS-40-e70178-s001.docx]

**Supplementary files**

***Supplementary Table 1.*** *Characteristics of the study sample: comparison between patients with sporadic SVD and CADASIL. A 95% confidence interval (CI) is provided for numerical data.*

| Variable | Sporadic SVD (n = 21) | CADASIL (n = 19) | P-value | 95% CI |
| --- | --- | --- | --- | --- |
| Age *M SD* | 73.6 ± 9.6 | 58.4 ± 8.41 | **< 0.001 ***** | [-0.87, -0.54] |
| Sex *(n, % male)* | 15 (71) | 13 (68) | 1 | - |
| Ethnicity *(n, %)*  Asian  White | 2 (10)  19 (90) | 1 (5)  18 (95) | 1 | - |
| Years education *M SD* | 14.9 ± 4.28 | 14.1 ± 3.12 | 0.672 | [-0.42, 0.28] |
| Diagnosis of depression (past & current) *(n, %)* | 6 (29) | 8 (42) | 0.57 | - |
| Diagnosis of depression (current) *(n, %)* | 4 (19) | 8 (42) | 0.21 | - |
| mRS median | 1 | 1 | 0.65 | [-0.42, 0.27] |
| MoCA total* | 24.2 ± 2.88 | 22.6 ± 6.27 | 0.929 | [-0.37, 0.34] |
| BMET total**  BMET executive  BMET memory | 12.9 ± 2.83  *7.14* ± *1.32*  *5.62* ± *2.11* | 11.7 ± 4.25  *6.28* ± *2.22*  *5.5* ± *2.43* | 0.607  *0.229*  *0.797* | [-0.44, 0.26]  *[-0.52, 0.15]*  *[-0.40, 0.31]* |
| Apathy (*n, %)* | 8 (38) | 12 (63) | 0.21 | **-** |
| AES Clinician | 33.7 ± 9.66 | 41.2 ± 13.9 | 0.11 | [-0.06, 0.58] |
| AES Self-rated | 31.8 ± 7.64 | 38.6 ± 10.6 | **0.03 *** | [0.08, 1.38] |
| AES Informant | 33.6 ± 8.22 | 41.9 ± 12,8 | **0.03 *** | [0.08, 0.67] |
| BDI | 8.38 ± 6.34 | 11.7 ± 8.96 | 0.303 | [-0.17, 0.51] |
| SF-36 (Mental) | 49.1 ± 9.29 | 46.2 ± 12.2 | 0.42 | [-0.89, 0.37] |
| SF-36 (Physical) | 35.2 ± 12.2 | 36.9 ± 10.5 | 0.64 | [-0.47, 0.77] |

** The MoCA total score was available for 38 participants out of 40 as explained in the Methods – Missing data.*

***The BMET total score was available for 39 participants out of 40 as explained in the Methods – Missing data.*

***Supplementary Table 2.*** *Multiple linear regressions assessing apathy in relationship to caregivers’ outcomes – additionally corrected for current depression status of patient.*

| Outcome variable | Unstandardised coefficient (β) of predictor;  [95% CI] | P-value |
| --- | --- | --- |
| NPI distress apathy | AES-C 0.12 [0.09; 0.154] | **< 0.001 ***** |
| NPI distress depression | AES-C 0.07 [0.023; 0.11] | **< 0.001 ***** |
| ZBI | AES-C 0.7 [0.29; 1.41] | **0.002 **** |
| HADS anxiety | AES-C 0.14 [-0.002; 0.29] | 0.05 |
| HADS depression | AES-C 0.13 [0.017; 0.25] | **0.03 *** |

***Supplementary Table 3.*** *Multiple linear regressions assessing apathy in relationship to patients’ outcomes – additionally corrected for current depression status of patient.*

| Outcome variable | Unstandardised coefficient (β) of predictor  and significant covariates; [95% CI] | P-value |
| --- | --- | --- |
| SF-36 MCS | AES-C -0.5 [-0.78; -0.21] | **0.001 **** |
| BDI | AES-C 0.42 [0.21; 0.64]  BMET total 0.79 [0.07; 1.51] | **< 0.001 *****  **0.013 *** |
